# Oxidative Stress and Inflammation as Targets for Novel Preventive and Therapeutic Approaches in Non-Communicable Diseases II

**DOI:** 10.3390/antiox11050824

**Published:** 2022-04-23

**Authors:** Chiara Nediani, Monica Dinu

**Affiliations:** 1 Department of Experimental and Clinical Biomedical Sciences “Mario Serio”, University of Florence, 50134 Florence, Italy; 2 Department of Experimental and Clinical Medicine, University of Florence, 50134 Florence, Italy; monica.dinu@unifi.it

Non-communicable diseases (NCDs) are non-infectious chronic pathologies—including obesity, metabolic syndrome, chronic kidney disease (CKD), cardiovascular (CV) diseases, cancer, and chronic respiratory diseases—which represent the main cause of death and disability for the general population [1]. Their growing prevalence is related to the increasing age of the population; urbanization; and lifestyle changes [1]. As previously reported, oxidative stress and inflammation induce and modulate several signaling pathways that play a crucial role in the pathophysiology and progression of these diseases [2]. Thus, they represent a good target for the development of several therapeutic strategies. This Special Issue consists of 12 articles providing different approaches to elucidate the underlying pathogenesis and treatment mechanisms of conditions related to oxidative stress and inflammation.

In the review by Fibbi et al. [3] the authors explore the role of oxidative stress in both osmolality-dependent and -independent impairment of cell and tissue functions observed in hyponatremic conditions. Hyponatremia is defined as a serum sodium concentration ([Na+]) < 136 mEq/L and has been associated with augmented morbidity and mortality. Following the description of neurological and systemic manifestations even in mild and chronic hyponatremia, the authors show how reduced extracellular [Na+] is associated with detrimental effects on cellular homeostasis independently of hypoosmolality, and how most of these alterations are elicited by oxidative stress. They also review a range of basic and clinical research showing that oxidative stress is a common denominator of degenerative processes linked to aging, neurocognitive deficits, osteoporosis, and cancer progression. Given all the evidence indicating that hyponatremia plays a part in exacerbating multiple manifestations of senescence and decreasing survival in cancer patients, they conclude by stressing the need for further studies to fully elucidate the specific molecular pathways triggered by reduced extracellular [Na+] and responsible for oxidative damage. Alemany-Cosme et al. [4] on the other hand, summarize the main findings regarding the oxidant and antioxidant mechanisms involved in Crohn’s disease (CD), their role in the immunological response, the environment’s effects on oxidative stress status, and its involvement in epigenetic changes/modifications. To date, it is known that in CD, oxidative stress is present not only locally in the most affected tissues, but also at a systemic level, and is associated with an unbalanced immune response and dysbiosis. This review highlights the need for further studies to determine environmental- and oxidative-stress-induced epigenetic changes that may contribute to the onset and development of CD.

Conditions causing predisposition to pro-inflammatory state and oxidative stress include obesity, which stimulates adipose tissue to release inflammatory mediators such as tumor necrosis factor (TNF)-α and interleukin (IL)-6 [5]. Obesity is also an important risk factor for breast cancer [6]. In the study by Martinez-Bernabe et al. [7] authors examined the effects of obesity-related inflammation on mitochondrial functionality in breast cancer cell lines and breast tumors, focusing on estrogen receptors (ER) ratio. The analysis of mitochondrial activity after 17β-estradiol, leptin, IL-6, and TNF-α exposure, which aimed to stimulate the hormonal conditions of a postmenopausal obese woman, showed that ER-β maintained mitochondrial functionality and avoided invasiveness in breast-cancer cell lines. Moreover, the authors found a strong correlation between IL-6 receptor gene expression and inflammation, mitochondrial functionality, and oxidative stress markers, as well as with ER-β. Overall, these findings confirm that under an obesity-related inflammation condition, the presence of ER-β allows the maintenance of mitochondrial functionality, reduced production of ROS, and high expression of antioxidant enzymes which result in a less aggressive phenotype.

The presence of high levels of ROS is also a hallmark of idiopathic pulmonary fibrosis (IPF), mainly due to the H_2_O_2_-generating enzyme NADPH oxidase 4 (NOX4) [8]. In turn, H_2_O_2_ is a substrate of the di-tyrosine peroxidase (DT) cross-linking, which appears to be involved in many diseases, including IPF. Recent studies have documented a significant increase in DT in IPF, but whether DT is formed in the lungs of IPF patients, and how its levels and localization contribute to the IPF pathogenesis [9], is unknown. In the study by Blaskovic et al. [10] authors wanted to deepen the role of DT and NOX4 in IPF with the perspective to find new therapies for IPF, since those in current use (Nintedanib and Pirfenidone) have adverse effects. They performed immunohistochemical staining for DT and NOX4 in pulmonary tissue from patients with IPF and controls. In IPF, both DT and NOX4 were present, whereas in the healthy lung DT showed little or no staining, and NOX4 was present mainly in normal vascular endothelium. The link between NOX4 and DT was addressed in MRC5 lung fibroblasts deficient in NOX4 activity (mutation in the CYBA gene). Induction of NOX4 by transforming growth factor beta 1 (TGFβ1) in fibroblasts led to moderate DT staining after the addition of a heme-containing peroxidase in control cells, but not in the fibroblasts deficient for NOX4 activity. These results indicate that DT is a histological marker of IPF and that NOX4 can generate enough H_2_O_2_ for DT formation in vitro. On the other hand, the absence of NOX4 and DT in all lung regions suggests that NOX4-dependent DT formation could be limited to the fibrotic foci.

To discover new biomarkers for the progression of CKD, a systemic disease to which development chronic oxidative stress and inflammation contribute, Vida et al. [11] analyzed several redox state markers in plasma of advanced CKD patients and isolated peripheral polymorphonuclear (PMNs) and mononuclear (MNs) leukocytes. Study patients were divided into healthy controls, non-dialysis-dependent-CKD (NDD-CKD) patients, hemodialysis (HD) and peritoneal dialysis (PD) patients and were characterized for the presence of some co-morbidities, for the etiology of CKD and for the treatment received. The analysis revealed increased oxidative stress and damage in plasma, PMNs and MNs from NDD-CKD, HD and PD patients compared to controls. Interestingly, PD patients showed greater oxidative stress than HD patients, especially in MNs. Based on these results, the authors encourage the evaluation of PMNs and MNs in CKD patients to follow both CKD progression and dialysis procedures.

In animal model studies, several approaches have been used to assess the damage induced by oxidative stress. Chang et al. [12] used a rodent model of stroke to demonstrate that glycerol is capable of alleviating post-stroke brain injury and associated acute kidney injury (AKI). The authors evaluated blood–brain barrier (BBB) integrity parameters and revealed that glycerol was useful in alleviating BBB disruption and reducing hemorrhagic stroke brain damage. In addition, they showed an improvement in kidney markers and a decrease in stress hormones levels after glycerol injection. Finally, they analyzed morphological alteration induced by hemorrhagic stroke in kidney structure and discovered a glycerol-induced modulation on cytokine-induced neutrophil chemoattractant 1 (CINC-1) and malondialdehyde (MDA), two urinary markers of oxidative stress overexpressed in AKI. Lazar et al. [13] also focused on kidney function, but in type I and II diabetes. In their study, they investigated the possibility of treating the manifestations of diabetic kidney disease (DKD) by inhibiting histone acetyltransferase p300/CBP, which regulates many genes and may be responsible for Nox expression, ROS production, inflammation and fibrosis, all manifestations of DKD. They used STZ-induced diabetic mice treated with C646 and showed a significant decrease in H3K27ac—an epigenetic mark of active gene expression, reduced ROS production and reduced inflammation-driving molecules and mesangial extracellular matrix (ECM) components responsible for fibrosis. Furthermore, using human embryonic kidney cells, they found a down-regulation of the luciferase level and a decrease in glomerular hypertrophy. All these data confirm the implication of p300/CBP in DKD and reveal the possibility of using a p300/CBP inhibitor to alleviate DKD.

Two other articles in this Special Issue used mouse models, in this case to assess cardiac function and the role of inflammation in the development of hypertension. Liu et al. [14] used wild-type (WT) and p47^phox^ knockout (KO) mice to investigate p47^phox^-dependent oxidant signaling in Angiotensin II (AngII) infusion-induced cardiac hypertrophy and cardiomyocyte apoptosis. In fact, the signaling pathways of p47^phox^ in the heart are still unclear. In their experiment, the authors showed how AngII infusion resulted in high blood pressure and cardiac hypertrophy in WT mice, whereas these pathological changes were significantly reduced in p47^phox^ KO mice. These findings confirm that p47^phox^ is a key player in mediating the AngII-induced oxidative stress signaling cascade from the phosphorylation of ASK1, MKK3/6 and MAPKs to the activation of H2AX and p53 and suggest that targeting p47^phox^ could have great therapeutic potential for preventing or treating AngII-induced cardiac dysfunction and damages. On the other hand, Sun et al. [15] demonstrated that an increase in endogenous μ-opioids in the nucleus tractus solitarius (NTS) induces a neurotoxicity cascade with enhanced Ang II binding to the AT1R receptor and activates the microglia which induces superoxide production. Furthermore, they showed how the increase in endogenous μ-opioids induces the formation of μOR/AT1R heterodimers and the TLR4-dependent inflammatory response, which attenuate the nitric oxide (NO)-dependent depressor effect. These results imply an important link between neurotoxicity and superoxide and deepen our understanding of μOR as a novel candidate for intervention in hypertensive conditions.

The search for antioxidants to be used in therapy and prevention of NCDs also relies on plant compounds. In this respect, oleuropein—a phenolic compound found in *Olea europaea* L. fruits and leaves—is one of the most studied bioactive compounds in the context of the Mediterranean diet. In previous studies, oleuropein exhibited a wide range of antioxidant, anti-inflammatory, antidiabetic, neuro- and cardioprotective, antimicrobial and immunomodulatory activities [16,17]. Furthermore, it was able to reduce crypt dysplasia in a short-term colon carcinogenesis experiment in rats and showed protective effects in colitis-associated colorectal cancer (CRC) in mice, suggesting that this molecule may reduce colon tumorigenesis [18]. However, whether these protective effects can be extended to already developed colon tumors it is not yet known. Ruzzolini et al. [19] evaluated the effect of oleuropein-rich leaf extracts (ORLE) for the first time in already-developed colon tumors arising in Apc (adenomatous polyposis coli) -mutated PIRC rats. The authors assessed whether one-week low-dose treatment with an ORLE-enriched diet could exert a beneficial effect against established colon cancer lesions and local and systemic inflammation. Although in vivo experiments were performed with a limited number of PIRC rats fed with ORLE, the overall results disclose a significant increase in tumor apoptosis together with a downregulation of proliferation associated with the inhibition of NO and relative pro-inflammatory mediators expressed by tumor cells and inflammatory cells of the tumor microenvironment. These findings suggest the possibility of testing ORLE as a complementary therapy in combination with standard anticancer drugs.

Columbianadin (CBN), another plant compound, was also tested. It is a natural coumarin isolated from *Angelica decursiva*, which has showed anticancer and platelet-aggregation-inhibiting properties. In their study, Jayakumar et al. [20] demonstrated that CBN exhibits compelling anti-inflammatory and hepatoprotective effects by preventing free-radical formation and decreasing the expression of mitogen-activated protein kinase (MAPK), followed by the suppression of the nuclear factor kappa B (NF-κB) pathways. This, in turn, led to inhibition of NO, inducible nitric oxide synthase (iNOS), TNF-α, and IL-1β in LPS-activated RAW cells and mouse liver. Although further study of the principal mechanisms is required, these data suggested that CBN represents a valid anti-inflammatory and hepatoprotective agent, and thus is a valid candidate for treating inflammation-mediated diseases. Finally, Li et al. [21] explored the effects of rebaudioside A (Reb A), a natural non-nutritive sweetener obtained from the extracts of *Stevia rebaudiana*. In previous studies, this compound has demonstrated many biological properties, such as anti-inflammatory, antioxidant, antifibrotic and anticancer properties [22]. In their study, Li et al. [21] assessed the effect of Reb A on the health span and lifespan of nematodes and investigated the potential mechanisms underlying Reb A-induced metabolic changes using a combination of transcriptomics and lipidomic approaches. In the model organism *C. elegans*, Reb A prolonged lifespan, enhanced oxidative stress resistance, and improved lipid metabolism. These findings serve as a ground for future studies exploring the potential medicinal and beneficial effects of Reb A as a replacement for caloric sugars in human foods and beverages.

The Guest Editors would like to thank all the authors, the reviewers who contributed to the success of this Special Issue, and the *Antioxidants* team for their valuable and constant support.
